# Dialysis or kidney transplantation in older adults? A systematic review summarizing functional, psychological, and quality of life-related outcomes after start of kidney replacement therapy

**DOI:** 10.1007/s11255-022-03208-2

**Published:** 2022-05-06

**Authors:** Tessa S. Schoot, Namiko A. Goto, Rob J. van Marum, Luuk B. Hilbrands, Angèle P. M. Kerckhoffs

**Affiliations:** 1 Department of Nephrology, Radboud University Medical Center, Radboud Institute for Health Sciences, Nijmegen, the Netherlands; 2 Department of Nephrology, Jeroen Bosch Hospital, ‘s-Hertogenbosch, the Netherlands; 3 Department of Geriatric Medicine, Jeroen Bosch Hospital, ‘s-Hertogenbosch, the Netherlands; 4 Department of Geriatric Medicine, University Medical Center Utrecht, Utrecht, the Netherlands; 5 Department of Clinical Pharmacology, Jeroen Bosch Hospital, ‘s-Hertogenbosch, the Netherlands; 6 Department of Elderly Care Medicine, Amsterdam University Medical Center, Amsterdam, the Netherlands

**Keywords:** Renal transplantation, Dialysis, Geriatric nephrology, Quality of life, Functional status

## Abstract

**Background:**

In older patients, the choice between kidney transplantation (KT) and dialysis may be complicated because of a high prevalence of comorbidities and geriatric syndromes. Ideally, this decision-making process focusses on older patients’ outcome priorities, which frequently include functional, psychological, and quality of life (QOL)-related outcomes.

**Purpose:**

This systematic review aims to summarize functional, psychological (including cognition), and QOL-related outcomes after start of kidney replacement therapy (KRT) in older adults.

**Methods:**

We searched PubMed and Embase for research that investigated change in these variables after start of KRT in patients aged ≥ 60 years. Data were extracted using the summary measures reported in the individual studies. Risk of bias was assessed with the ROBINS-I tool.

**Results:**

Sixteen observational studies (prospective *n* = 9, retrospective *n* = 7; KT-recipients *n* = 3, dialysis patients *n* = 13) were included. The results show that QOL improves in the majority of the older KT recipients. After start of dialysis, QOL improved or remained stable for most patients, but this seems less prevalent than after KT. Functional status decreases in a substantial part of the older dialysis patients. Furthermore, the incidence of serious fall injuries increases after start of dialysis. Nutritional status seems to improve after start of dialysis.

**Conclusion:**

The interpretability and comparability of the included studies are limited by the heterogeneity in study designs and significant risk of bias in most studies. Despite this, our overview of functional, psychological (including cognition), and QOL-related outcomes is useful for older adults and their clinicians facing the decision between KT and dialysis.

**Supplementary Information:**

The online version contains supplementary material available at 10.1007/s11255-022-03208-2.

## Introduction

Older patients with end-stage kidney disease (ESKD) can be treated with kidney replacement therapy (kidney transplantation (KT) and dialysis) or they can choose conservative care. Of the two kidney replacement therapy (KRT) modalities, KT is usually preferred [1–3] because, in general, KT recipients have superior survival [4–6] and better quality of life (QOL) [7] compared to patients treated with dialysis. However, for older adults who are eligible for both KRT modalities, KT is not always superior to dialysis. Factors such as older age, comorbidities, and geriatric syndromes (e.g. cognitive impairment, frailty, and falls) are associated with a higher risk of complications and mortality after KT [1, 5, 6, 8, 9].

For individual older patients who want to be treated with KRT, the risks and benefits of KT should therefore be weighed against the risks and benefits of dialysis [1]. In this process, the individual patient’s outcome priorities should play an important role. Many older patients with ESKD consider outcomes related to functional status, psychological and cognitive status, and quality of life (QOL) more important than traditional medical outcomes such as mortality or graft survival [10–13]. For example, 75% of the older adults with ESKD considers ‘maintaining independence’ as one of the most important health outcomes [11]. However, in the current decision-making process regarding KT and dialysis, the focus lies on traditional medical outcomes instead of these functional, psychological, and QOL-related outcomes [1–3].

This systematic review aims to summarize data about functional, psychological (including cognition), and QOL-related outcomes after KT and dialysis in older adults. The results of this review can support older adults and their clinicians in the decision-making process concerning the choice between KT and dialysis.

## Methods

We prospectively registered the study protocol in the International Prospective Register of Systematic Reviews (PROSPERO) [14] and followed the Preferred Reporting Items for Systematic Reviews and Meta-Analyses (PRISMA) guideline [15]. Appendix 1 and Appendix 2 show the completed PRISMA checklists (general version and abstract version, respectively).

### Selection of outcome variables

We first composed a list of all potentially relevant functional, psychological (including cognition), and QOL-related outcomes that are especially relevant for the geriatric population by performing an extensive literature search focusing on these outcomes, the expectations, and the outcome priorities of older patients with advanced stages of chronic kidney disease, older KT recipients, and older dialysis patients. We searched relevant clinical guidelines, QOL questionnaires, the core outcomes identified by the Standardised Outcomes in Nephrology (SONG) initiative [16], and used our clinical experience (NA, AK and RM are geriatricians, LH and AK are nephrologists, and TS is resident in internal medicine). Duplicates were removed and similar outcome measures were grouped together. We then selected those outcome measures that we considered to be relevant and operationalizable for older adults facing the decision-making between KT and dialysis. Table 1 describes the 12 outcome measures that are included in this systematic review, which are QOL, functional status, frailty, falls, nutritional status, body mass index (BMI), mood and anxiety disorders, cognition, delirium, incontinence, polypharmacy, and sarcopenia.

**Table 1:** Geriatric and quality of life-related outcomes included in the systematic review

Outcome variable	Description	Number of studies matching eligibility criteria
Quality of life (QOL)	QOL questionnaires, health-related QOL, self-rated health	5
Functional status	Gait speed, grip strength, measures of mobility and balance, use of walking aid, level of exercise, activities of daily living (ADL), instrumental ADL (iADL), living situation	8
Frailty	Frailty (indexes)	2
Falls	Falls, fractures	2
Nutritional status	Nutritional status, malnutrition tests	2
Body mass index (BMI)	Weight, BMI	2
Mood and anxiety disorders	Mood (disorder) tests, anxiety (disorder) tests, depression, dysthymia, anxiety disorders	2
Cognition	Cognitive function tests, dementia, cognitive impairment	1
Delirium	Delirium	0
Incontinence	Incontinence (urinary or fecal)	0
Polypharmacy	Number of drugs, polypharmacy	0
Sarcopenia	Sarcopenia, muscle strength, muscle mass	0

### Inclusion and exclusion criteria

We included longitudinal, original research that investigated one or more of the outcome measures listed in Table 1 after start of kidney replacement therapy (i.e. KT, hemodialysis (HD), or peritoneal dialysis (PD)) in patients aged 60 years or older. We excluded studies without a baseline measurement of the investigated outcome measure. Only publications in English were included. Studies with critically ill patients or recipients of multiple organ transplants were excluded.

### Search strategy

Appendix 3 shows the search strategy. In short, we searched PubMed and Embase using the terms ‘older adults’, ‘kidney transplantation’, ‘dialysis’, ‘cognition’, ‘mood disorder’, ‘frailty’, ‘nutritional status’, ‘functional status’, ‘sarcopenia’, ‘quality of life’, ‘polypharmacy’, and ‘incontinence’, and multiple synonyms of these terms. No limits were used in the PubMed search. In the Embase search, we used a filter to exclude conference abstracts. The last search was conducted in November 2021.

### Study selection

The search results were screened by title and abstract by one researcher (TS) using the web-based app Rayyan [17]. Next, the full texts of papers that potentially met the eligibility criteria were assessed independently and blinded by two researchers (NG, TS). Disagreements between the reviewers were resolved by consulting another researcher (AK).

### Data extraction

We combined papers that reported results from the same study. Data extraction was performed by one researcher (TS). Extracted data concerned quantitative data on study characteristics, population, and the outcomes of interest. Data on all results that were compatible with one of the predefined outcomes (Table 1) was extracted. We used the summary measures reported in the individual studies instead of one principal summary measure (e.g. incidence) because of the expected heterogeneity across studies.

### Bias assessment

Bias assessment was performed by one investigator (TS) using the Risk Of Bias In Non-randomized Studies of Interventions (ROBINS-I) tool [18]. First, the risk of bias was judged in the seven subdomains of ROBINS-I and, subsequently, the overall risk of bias was judged. The risk-of-bias plots were created with the web app Robvis [19].

## Results

### Search results

**Fig. 1:**
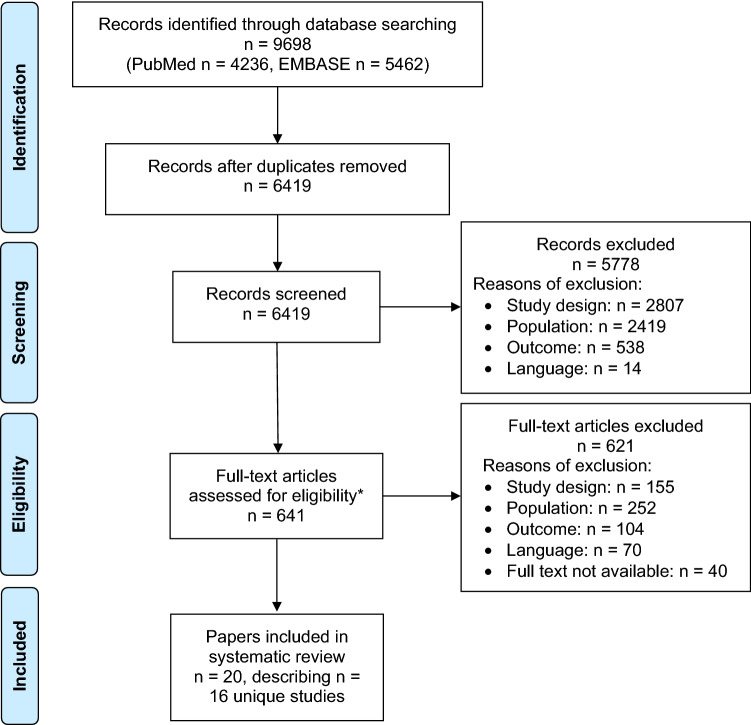
Flow diagram of database search and study selection. *Inter-rater agreement level 98%

Figure 1 shows the study selection process. Database search revealed 6419 unique records. After title/abstract screening, 641 papers remained for full-text assessment. Of these, 20 papers matched the eligibility criteria. Inter-rater agreement was 98%. After combining papers that described the results of the same study (i.e. Goto et al., Goto et al., and Van Loon et al. [20–22], Heldal et al. and Lønning et al.[23, 24], and Bowling et al. and Plantinga et al. [25, 26]), 16 unique studies remained.

### Study characteristics

Appendix 4 shows the study characteristics of the included studies. All studies were observational studies (prospective *n* = 9; retrospective *n* = 7). They originated from Asia, Europe, and Northern America (*n* = 3, *n* = 7, and *n* = 6, respectively), and all but one study [27] were published within the last 20 years. Three studies included KT recipients [23, 24, 28, 29] and thirteen studies included dialysis patients (only HD *n* = 4 [25, 26, 30–32]; only PD *n* = 2 [27, 33]; both HD and PD *n* = 6 [20–22, 34–38]; not specified *n* = 1 [39]). None of the studies included both KT and dialysis patients. Study and population characteristics varied markedly (see Appendix 4). For example, the sample size varied from 12 to 81,653 patients, follow-up duration varied from several weeks to more than 2 years, and minimum age limit varied from 60 to 80 years.

### Risk of bias

**Fig. 2:**
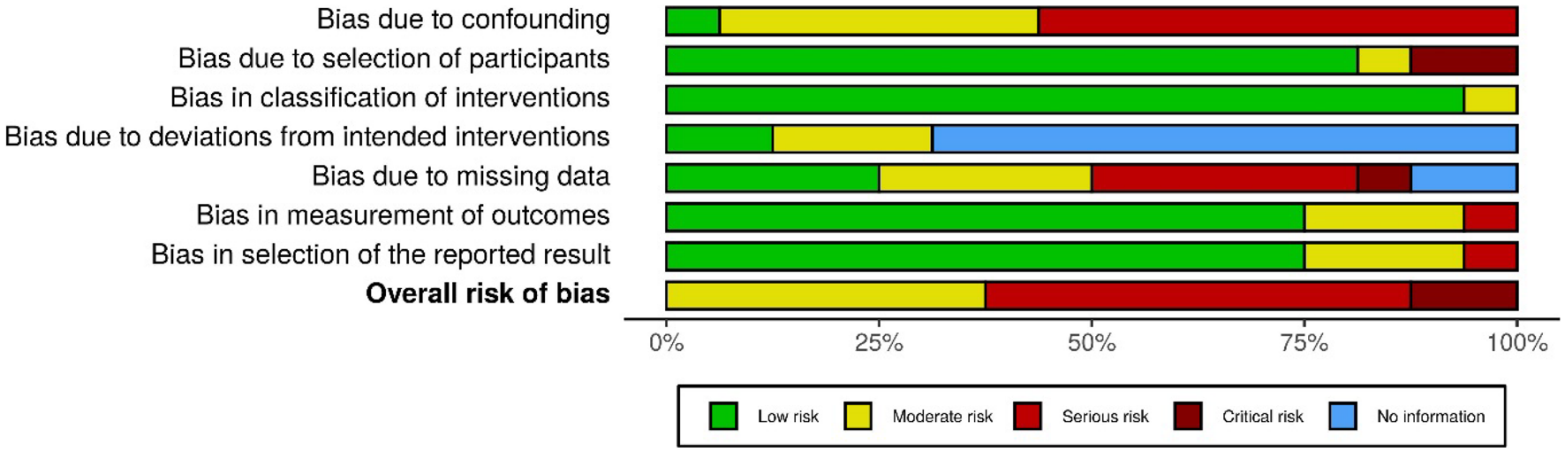
Bias assessment summary plot

**Fig. 3:**
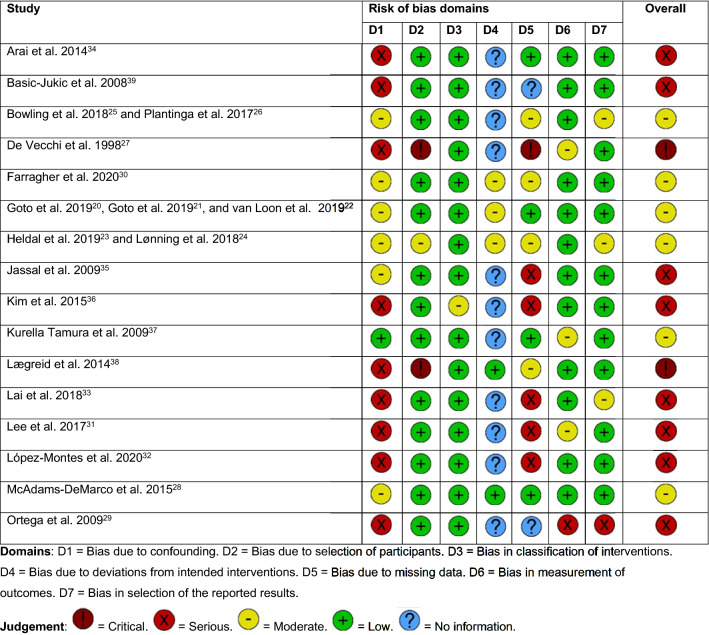
Traffic light plot: risk of bias per study

The overall risk of bias was moderate in six studies [20–26, 28, 30, 37], serious in eight studies [29, 31–36, 39], and critical in two studies [27, 38] (Figs. 2, 3). The risk of bias was highest in the domains ‘confounding’ and ‘missing data’. The majority of the studies had low risk of bias in the domains ‘selection of participants’, ‘classification of interventions’, and ‘selection of the reported results’.

### Outcomes

Table 1 shows the number of included studies per outcome variable. Five studies investigated more than one outcome variable. None of the studies that matched the eligibility criteria investigated delirium, incontinence, polypharmacy, or sarcopenia. In the following sections, the results are presented per outcome variable.

### Quality of life (QOL)

#### KT recipients (2 studies)

One single-center [23, 24] and one multicenter [29] prospective cohort study investigated QOL before and after KT in older adults (aged ≥ 65 years and > 60 years, respectively).

In the first study [23, 24], QOL was assessed with the kidney disease quality of life short form (KDQOL-SF), which is composed of a generic part (short form (SF)-36) and a kidney-disease specific part. Compared to baseline, mean overall QOL significantly improved two months after KT. Subsequently, mean overall QOL remained stable until the end of follow-up (i.e. one year after KT). Similar trajectories were seen in all QOL-subdomains, although not all changes were statistically significant. Participants were also asked to rate their total health on a numerical scale from 0 (worst possible) to 100 (best possible). This ‘total health’ score also improved two months after KT (increase from 56 to 67, *p* < 0.05), and improved even more six months after KT (to 70, *p* < 0.05 compared to baseline).

Similar results were found in the multicenter study [29]. This study used the SF-36 and EuroQol-5D questionnaire to assess QOL. EuroQol-5D measures QOL in five general domains (mobility, self-care, usual activities, pain/discomfort, and anxiety/depression). The summary scores of both questionnaires improved one year after KT compared to baseline. However, statistical analyses were not performed and the study has a serious risk of bias.

#### Dialysis patients (3 studies)

Two relatively large multicenter studies [20–22, 36] (*n* = 192 and *n* = 410, respectively) investigated QOL before and after start of dialysis. The first study [20–22] used the EuroQol-5D questionnaire to assess QOL. Six months after start of dialysis, mean QOL was not significantly different compared to baseline (change in mean summary score + 0.03, *p* = 0.10; change in visual analogue scale score + 0.3, *p* < 0.01, but not considered clinically significant). In patients who survived for more than 6 months, QOL had improved in 44.4%, remained stable in 31.4% and declined in 24.2% of the patients.

The second multicenter study [36] used the KDQOL-36 questionnaire, which measures QOL in five domains (physical function, mental function, burden of kidney disease, symptoms and problems of kidney disease, and effect of kidney disease on daily life). One year after start of dialysis, QOL had improved in four of the domains. However, absolute scores were not reported, relevant statistical analyses were not performed, and this study has a serious risk of bias.

In addition, one small single-center study [33] (*n* = 29) investigated QOL before and after start of PD. QOL was assessed with the KDQOL-SF. Results were only reported for the subdomain ‘cognitive function’, in which the mean score improved from 90 at baseline to 91 two years after start of PD. Statistical analyses were not performed and the risk of bias of the study is serious.

### Functional status

#### KT recipients (0 studies)

No data available.

#### Dialysis patients (8 studies)

Change in functional status after start of dialysis was investigated in one large database study (*n* = 3702, HD and PD patients residing in a nursing home) [37], one multicenter prospective cohort study (*n* = 187, HD and PD patients) [20–22], two single-center prospective cohort studies (*n* = 117 and *n* = 46, HD patients only) [31, 32] and two single-center retrospective cohort studies (*n* = 63, PD patients only; *n* = 97 HD and PD patients) [27, 35]. Functional status was assessed with several different measurement instruments: the Katz activities of daily living (ADL) questionnaire [20–22, 31, 32], the Lawton and Brody instrumental activities of daily living (IADL) questionnaire [20–22, 31, 32], a summary measure composed of the ADL and IADL score [20–22], the Short Physical Performance Battery (SPPB) [32], the Karnofsky Performance Score (KPS) [27], the Minimum Data Set-ADL (MDS-ADL) [37], and change in living situation [35].

Two studies found that mean functional status worsened one year after start of dialysis compared to baseline [27, 32, 37]. Another study reports that the percentage of patients living in a full-time care setting or nursing home also increased [35]. In the largest cohort study [20–22], functional status worsened in 43.4% of the patients who were still alive at six months after start of dialysis. Functional status remained stable in 37.2% and improved in 19.4% of the patients. In multivariable analysis, the composite outcome of functional decline and death was associated with older age and frailty.

There is only one small study [31] (*n* = 46) in which functional status did not significantly change after start of dialysis (follow-up one year). However, follow-up measurements were performed in only 29 patients (60%) and this study has a serious risk of bias.

Two single-center retrospective cohort studies investigated the change in functional status in institutionalized patients [30, 34]. The first study [34] included patients (*n* = 202, age > 75 years) who were hospitalized at start of HD or PD. At hospital discharge, functional status had declined in 69% of the patients. The other study [30] included older patients (*n* = 449, age > 60 years) at admission to a rehabilitation center after start of HD. At discharge from the rehabilitation center (median 43 days), functional status had improved in 95% of the patients.

In summary, these studies indicate that start of dialysis is often associated with a decrease in functional status.

### Frailty

#### KT recipients (1 study)

One prospective cohort study investigated change in the level of frailty after KT [28]. In this study, frailty was assessed with the Fried frailty score, which consists of five components (weight loss, low grip strength, exhaustion, low activity, and slow walking speed) that are scored as 0 (absent) or 1 (present). In the subgroup of patients aged ≥ 65 years, frailty score improved 3 months after KT (mean change -0.3; no statistical analyses reported). However, it is unclear whether this change is clinically relevant.

#### Dialysis patients (1 study)

For dialysis patients, one small prospective cohort study investigated change in the level of frailty [31]. Participants (*n* = 46) were HD patients aged 65 years or older. Frailty was assessed with a comprehensive geriatric assessment, which included nine items (presence of malignancy, number of comorbidities, serum albumin level, ADL score, IADL score, presence of dementia, risk of delirium, presence of malnutrition, and midarm circumference). Frailty was defined as a score of ≥ 10. One year after start of HD, the level of frailty had decreased (decrease from 8 to 6, *p* < 0.001), which largely relied on improved nutritional status. It is unclear whether this change in score is clinically relevant.

### Falls

#### KT recipients (0 studies)

No data available.

#### Dialysis patients (2 studies)

One large database study (*n* = 81,654, HD patients only) [25, 26] and one larger multicenter prospective cohort study (*n* = 160, HD and PD patients) [20–22] investigated change in the incidence of falls after start of dialysis. The database study investigated serious fall injury (defined as a fall causing a fracture, brain injury, or joint dislocation). The incidence of serious fall injury was higher in the year after initiation of HD compared to the year before (cumulative incidence 7.6% vs. 3.6%; incidence rate 108/1000 patient years vs. 64/1000 patient years). The multicenter study investigated accidental falls. The cumulative incidence of accidental falls was the same in the six months before and the six months after start of dialysis (both 26%) [20–22].

### Nutritional status

#### KT recipients (0 studies)

No data available.

#### Dialysis patients (2 studies)

Two cohort studies assessed nutritional status, both with a questionnaire (mini nutritional assessment and subjective global assessment, respectively), before and after start of dialysis [27, 31]. The first study included PD patients aged 70 years and older [27], while the second study included HD patients aged 65 years and older [31]. Both studies report that nutritional status improved after start of dialysis.

### Body mass index (BMI)

#### KT recipients (0 studies)

No data available.

#### Dialysis patients (2 studies)

One database study (*n* = 233, HD and PD patients) [38] and one small single-center prospective cohort study (*n* = 12, dialysis modality not specified) [39] investigated change in body mass index (BMI) after start of dialysis. In the first study, mean BMI decreased after start of dialysis, while in the second study, BMI did not change. The risk of bias of these studies was judged as critical [38] and serious [39].

### Mood and anxiety disorders

#### KT recipients (0 studies)

No data available.

#### Dialysis patients (2 studies)

Two prospective cohort studies assessed change in depressive symptoms after start of dialysis [32, 36]. The largest study included 410 dialysis patients aged 65 years and older (HD and PD) [36]. The other study included 117 HD patients aged 70 years and older [32]. Depressive symptoms were evaluated with the Beck Depression Inventory (BDI) [36] and Geriatric Depression Scale (GDS) [32]. In both questionnaires, higher scores indicate more severe depressive symptoms. In HD patients, mean BDI score increased from 16 at baseline to 17 at 1 year after start of dialysis [36], while in the other study GDS score decreased from 7 at baseline to 5 at 1 year after start of dialysis [32]. In PD patients, mean BDI score decreased from 20 to 7 [36]. It is not stated whether these differences were statistically significant or clinically relevant. Moreover, both studies have a serious risk of bias.

### Cognition

#### KT recipients (0 studies)

No data available.

#### Dialysis patients (1 study)

Only one single-center prospective cohort study investigated changes in cognitive status in older dialysis patients [32]. This study included 117 HD patients aged 70 years and older. Cognitive status was assessed with the Mini Cognitive Examination of Lobo, which is a Spanish modification of the more well-known Mini Mental State Examination (MMSE). The median MCE score improved from 26 at baseline to 27 one year after start of HD. However, it is unclear whether this difference is statistically significant or clinically relevant.

## Discussion

This systematic review provides a comprehensive summary of the available evidence regarding functional, psychological/cognition, and QOL-related outcomes after start of kidney replacement therapy in older adults. The PRISMA guideline [15] was strictly followed, which ensures the methodological quality of this systematic review.

This systematic review has several interesting results. The available evidence shows that QOL of older adults usually improves after KT [23, 24, 29]. After start of dialysis, QOL can also improve, but this seems to be the case in a smaller amount of the patients than after KT [20–22, 33, 36]. This systematic review also shows that functional status decreases in a substantial proportion of older patients after start of dialysis [20–22, 27, 31, 32, 34, 35, 37]. Moreover, the incidence of serious fall injuries increases after start of dialysis [25, 26]. Health care professionals should be aware of this potential decline in functional status after start of dialysis in older adults. For selected patients, rehabilitation after start of dialysis might prevent functional decline [30].

However, this systematic review also reveals that there is few reliable data available about functional, psychological/cognition and QOL-related outcomes after start of kidney replacement therapy in older adults. Only sixteen studies matched our eligibility criteria. Of these, only three studies included KT recipients [23, 24, 28, 29], while studies comparing KT recipients with dialysis patients were not available at all. Remarkably, none of the included studies investigated delirium, incontinence, polypharmacy, or sarcopenia, and only one of the included studies investigated cognitive status. Of the sixteen available studies, the risk of bias was serious [29, 31–36, 39] or critical [27, 38] in ten of them, mostly due to a significant risk of bias due to confounding and/or missing data (Figs. 2, 3). None of the studies included a control group. On top of that, comparability of the included studies was limited by heterogeneity in study design (e.g. differences in measurement instrument(s), timing of measurements) and study population (e.g. differences in age, comorbidity burden, baseline functional status). Lastly, there were marked baseline differences between patient cohorts (e.g. KT recipients were, in general, less frail than dialysis patients). Moreover, not all dialysis patients in the included studies might have been eligible for KT as well. This limits the comparability of studies that included dialysis patients and studies that included KT recipients. To reduce the impact of selection bias, future studies aiming to compare outcomes of KT and dialysis in older adults should preferably include only patients who are eligible for both KT and dialysis.

One of the key steps of successful shared decision-making in older adults is the identification of patient’s values and treatment goals [40]. It is known that the goals of care of older patients are not limited to medical outcomes but also include outcomes in other domains, such as independence and autonomy, living accommodation, and mobility [41]. It has also been demonstrated that health care professionals poorly estimate the health outcome priorities of older patients with ESKD or other chronic diseases [11, 42, 43]. Thus, the findings of this systematic review, i.e. a summary of functional, psychological, and QOL-related outcomes, can support the (shared) decision-making process regarding the choice between dialysis and KT in older adults.

A limitation of our systematic review is that it might not capture all outcome measures that are relevant for the geriatric population. For example, we have not included ‘having social support’, while previous research into the perspectives on what matters most to older hemodialysis patients identified this as a major theme [44]. It should also be acknowledged that traditional medical outcomes, such as life expectancy and symptoms (like fatigue), can also be relevant to older adults facing the decision between KT and dialysis [16, 44–46].

Future research in this field will face two methodological challenges. First, since a randomized trial is neither ethical nor practical, future studies will inevitably be observational cohort studies as well. In future studies, the risk of bias due to confounding could be limited by including only patients that are eligible for both KT and dialysis. Another option is to perform target trial emulation, in which design principles from randomized trials are applied to observational data [47]. Second, the measurement of most functional, psychological, and QOL-related outcomes is prone to bias because gold standards are lacking and most measurements are not validated in the subgroup of older patients with ESKD. New QOL questionnaires for this subgroup might solve part of this problem [48].

## Conclusion

This systematic review provides a comprehensive summary of the available evidence regarding functional, psychological (including cognition), and QOL-related outcomes after start of kidney replacement therapy in older adults. The available evidence is scarce and has methodological limitations. Despite this, our results show that QOL improves in the majority of older KT recipients. In dialysis patients, the chance of improvement of QOL seems lower than after KT. Moreover, health care professionals should be aware of a potential decline in functional status after start of dialysis.

## Supplementary Information

Below is the link to the electronic supplementary material.

Supplementary file1 (DOCX 41 KB)
