# Supplementary material for: Dialysis or kidney transplantation in older adults? A systematic review summarizing functional, psychological, and quality of life-related outcomes after start of kidney replacement therapy
Source: Int Urol Nephrol. 2022 May 6;54(11):2891–900. doi: 10.1007/s11255-022-03208-2 (PMC9534800; doi:10.1007/s11255-022-03208-2)
Supplement: Supplementary file 1 — Supplementary file1 (DOCX 41 KB) [file 11255_2022_3208_MOESM1_ESM.docx]

**‘Dialysis or kidney transplantation in older adults? A systematic review summarizing functional, psychological, and quality of life-related outcomes after start of kidney replacement therapy’**

Tessa S. Schoot^1,2^, Namiko A. Goto^3,4^, Rob J. van Marum^3,5,6^, Luuk B. Hilbrands^1^, Angèle P.M. Kerckhoffs^2,3^

**Corresponding author**

Tessa S. Schoot, MD

Radboud University Medical Center, Radboud Institute for Health Sciences, Department of Nephrology, Nijmegen, the Netherlands

Jeroen Bosch Hospital, Department of Nephrology, ‘s-Hertogenbosch, the Netherlands

E-mail: [t.schoot@jbz.nl](mailto:t.schoot@jbz.nl)

ORCID ID: 0000-0001-5575-617X

**Table of contents**

Appendix 1. PRISMA checklist Page 2

Appendix 2. PRISMA abstract checklist Page 5

Appendix 3. Search strategy Page 6

Appendix 4. Study characteristics Page 9

**Appendix 1.** Prisma checklist

| **Section and Topic** | **Item #** | **Checklist item** | **Location where item is reported** |
| --- | --- | --- | --- |
| **TITLE** | | |  |
| Title | 1 | Identify the report as a systematic review. | Title |
| **ABSTRACT** | | |  |
| Abstract | 2 | See the PRISMA 2020 for Abstracts checklist. | Abstract |
| **INTRODUCTION** | | |  |
| Rationale | 3 | Describe the rationale for the review in the context of existing knowledge. | Introduction |
| Objectives | 4 | Provide an explicit statement of the objective(s) or question(s) the review addresses. | Introduction |
| **METHODS** | | |  |
| Eligibility criteria | 5 | Specify the inclusion and exclusion criteria for the review and how studies were grouped for the syntheses. | Methods |
| Information sources | 6 | Specify all databases, registers, websites, organisations, reference lists and other sources searched or consulted to identify studies. Specify the date when each source was last searched or consulted. | Methods |
| Search strategy | 7 | Present the full search strategies for all databases, registers and websites, including any filters and limits used. | Supplementary text 1 |
| Selection process | 8 | Specify the methods used to decide whether a study met the inclusion criteria of the review, including how many reviewers screened each record and each report retrieved, whether they worked independently, and if applicable, details of automation tools used in the process. | Methods |
| Data collection process | 9 | Specify the methods used to collect data from reports, including how many reviewers collected data from each report, whether they worked independently, any processes for obtaining or confirming data from study investigators, and if applicable, details of automation tools used in the process. | Methods |
| Data items | 10a | List and define all outcomes for which data were sought. Specify whether all results that were compatible with each outcome domain in each study were sought (e.g. for all measures, time points, analyses), and if not, the methods used to decide which results to collect. | Methods + Table 1 |
|  | 10b | List and define all other variables for which data were sought (e.g. participant and intervention characteristics, funding sources). Describe any assumptions made about any missing or unclear information. | Methods |
| Study risk of bias assessment | 11 | Specify the methods used to assess risk of bias in the included studies, including details of the tool(s) used, how many reviewers assessed each study and whether they worked independently, and if applicable, details of automation tools used in the process. | Methods |
| Effect measures | 12 | Specify for each outcome the effect measure(s) (e.g. risk ratio, mean difference) used in the synthesis or presentation of results. | Methods |
| Synthesis methods | 13a | Describe the processes used to decide which studies were eligible for each synthesis (e.g. tabulating the study intervention characteristics and comparing against the planned groups for each synthesis (item #5)). | Methods |
|  | 13b | Describe any methods required to prepare the data for presentation or synthesis, such as handling of missing summary statistics, or data conversions. | Not applicable |
|  | 13c | Describe any methods used to tabulate or visually display results of individual studies and syntheses. | Not applicable |
|  | 13d | Describe any methods used to synthesize results and provide a rationale for the choice(s). If meta-analysis was performed, describe the model(s), method(s) to identify the presence and extent of statistical heterogeneity, and software package(s) used. | Methods |
|  | 13e | Describe any methods used to explore possible causes of heterogeneity among study results (e.g. subgroup analysis, meta-regression). | Not applicable |
|  | 13f | Describe any sensitivity analyses conducted to assess robustness of the synthesized results. | Not applicable |
| Reporting bias assessment | 14 | Describe any methods used to assess risk of bias due to missing results in a synthesis (arising from reporting biases). | Not applicable |
| Certainty assessment | 15 | Describe any methods used to assess certainty (or confidence) in the body of evidence for an outcome. | Not applicable |
| **RESULTS** | | |  |
| Study selection | 16a | Describe the results of the search and selection process, from the number of records identified in the search to the number of studies included in the review, ideally using a flow diagram. | Results + Figure 1 |
|  | 16b | Cite studies that might appear to meet the inclusion criteria, but which were excluded, and explain why they were excluded. | Not applicable |
| Study characteristics | 17 | Cite each included study and present its characteristics. | Results + Supplementary table 3 |
| Risk of bias in studies | 18 | Present assessments of risk of bias for each included study. | Results + Figure 2 + Figure 3 |
| Results of individual studies | 19 | For all outcomes, present, for each study: (a) summary statistics for each group (where appropriate) and (b) an effect estimate and its precision (e.g. confidence/credible interval), ideally using structured tables or plots. | Results |
| Results of syntheses | 20a | For each synthesis, briefly summarise the characteristics and risk of bias among contributing studies. | Supplementary table 3 + Figure 3 |
|  | 20b | Present results of all statistical syntheses conducted. If meta-analysis was done, present for each the summary estimate and its precision (e.g. confidence/credible interval) and measures of statistical heterogeneity. If comparing groups, describe the direction of the effect. | Not applicable |
|  | 20c | Present results of all investigations of possible causes of heterogeneity among study results. | Not applicable |
|  | 20d | Present results of all sensitivity analyses conducted to assess the robustness of the synthesized results. | Not applicable |
| Reporting biases | 21 | Present assessments of risk of bias due to missing results (arising from reporting biases) for each synthesis assessed. | Not applicable |
| Certainty of evidence | 22 | Present assessments of certainty (or confidence) in the body of evidence for each outcome assessed. | Not applicable |
| **DISCUSSION** | | |  |
| Discussion | 23a | Provide a general interpretation of the results in the context of other evidence. | Discussion |
|  | 23b | Discuss any limitations of the evidence included in the review. | Discussion |
|  | 23c | Discuss any limitations of the review processes used. | Not applicable |
|  | 23d | Discuss implications of the results for practice, policy, and future research. | Discussion |
| **OTHER INFORMATION** | | |  |
| Registration and protocol | 24a | Provide registration information for the review, including register name and registration number, or state that the review was not registered. | Methods |
|  | 24b | Indicate where the review protocol can be accessed, or state that a protocol was not prepared. | Methods |
|  | 24c | Describe and explain any amendments to information provided at registration or in the protocol. | Not applicable |
| Support | 25 | Describe sources of financial or non-financial support for the review, and the role of the funders or sponsors in the review. | Funding |
| Competing interests | 26 | Declare any competing interests of review authors. | Disclosures |
| Availability of data, code and other materials | 27 | Report which of the following are publicly available and where they can be found: template data collection forms; data extracted from included studies; data used for all analyses; analytic code; any other materials used in the review. | Not applicable |

**Appendix 2.** PRISMA abstract checklist

| **Section and Topic** | **Item #** | **Checklist item** | **Reported (Yes/No)** |
| --- | --- | --- | --- |
| **TITLE** | | |  |
| Title | 1 | Identify the report as a systematic review. | Yes |
| **BACKGROUND** | | |  |
| Objectives | 2 | Provide an explicit statement of the main objective(s) or question(s) the review addresses. | Yes |
| **METHODS** | | |  |
| Eligibility criteria | 3 | Specify the inclusion and exclusion criteria for the review. | Yes |
| Information sources | 4 | Specify the information sources (e.g. databases, registers) used to identify studies and the date when each was last searched. | Yes |
| Risk of bias | 5 | Specify the methods used to assess risk of bias in the included studies. | Yes |
| Synthesis of results | 6 | Specify the methods used to present and synthesise results. | Yes |
| **RESULTS** | | |  |
| Included studies | 7 | Give the total number of included studies and participants and summarise relevant characteristics of studies. | Yes |
| Synthesis of results | 8 | Present results for main outcomes, preferably indicating the number of included studies and participants for each. If meta-analysis was done, report the summary estimate and confidence/credible interval. If comparing groups, indicate the direction of the effect (i.e. which group is favoured). | Yes |
| **DISCUSSION** | | |  |
| Limitations of evidence | 9 | Provide a brief summary of the limitations of the evidence included in the review (e.g. study risk of bias, inconsistency and imprecision). | Yes |
| Interpretation | 10 | Provide a general interpretation of the results and important implications. | Yes |
| **OTHER** | | |  |
| Funding | 11 | Specify the primary source of funding for the review. | No |
| Registration | 12 | Provide the register name and registration number. | No |

**Appendix 3.** Search strategy

**1. PubMed**

Complete search

((frail elderly [mesh] or geriatrics [mesh] or very-elder* [tiab] or elder* [tiab] or eldest [tiab] or geriatri* [tiab] or old age* [tiab] or oldest old* [tiab] or very old* [tiab] or senior* [tiab] or septuagenarian* [tiab] or nonagenarian* [tiab] or octogenarian* [tiab] or octagenarian* [tiab] or centenarian* [tiab] or centarian* [tiab] or supercentenarian* [tiab] or older people [tiab] or older subject* [tiab] or older patient* [tiab] or older adult* [tiab] or nursing home [tiab] or 60 year* [tiab] or 65 year* [tiab] or 70 year* [tiab] or 75 year* [tiab] or 80 year* [tiab] or 85 year* [tiab] or 90 year* [tiab] or 95 year* [tiab] or 100 year* [tiab]) AND (((Renal dialysis [mesh] or dialys* [tiab] or hemodialys* [tiab] or haemodialys* [tiab]) OR (Renal Replacement Therapy [Mesh] or renal replacement [tiab] or kidney replacement [tiab])) OR (Kidney transplantation [mesh] or kidney transplant* [tiab] or renal transplant* [tiab] or kidney graft* [tiab] or renal graft* [tiab]))) AND ((((((((("Cognitive Dysfunction"[Mesh] or cognit* [tiab] or MCI [tiab] or mental* [tiab] or neurocognit* [tiab] or "Mental Status and Dementia Tests"[Mesh] or MOCA [tiab] or MMSE [tiab] or dementia [mesh] or dement* [tiab] or Alzheimer [tiab] or "Delirium"[Mesh] or delirium* [tiab] or "Confusion"[Mesh] or disorientation [tiab] or confusion* [tiab]) OR ("Mood Disorders"[Mesh] or mood* [tiab] or affective [tiab] or "Depression"[Mesh] or depressi* [tiab] or neuros* [tiab] or neurot* [tiab] or "Psychotic Disorders"[Mesh] or psychosis [tiab] or psychoses [tiab] or psychotic [tiab] or Anxiety[Mesh] or Anxiety Disorders [Mesh] or anxiety [tiab])) OR ("Frailty"[Mesh] or frail* [tiab] or vulnerab* [tiab])) OR ("Body Weight"[Mesh] or weight [tiab] or "Weight Loss"[Mesh] or "Cachexia"[Mesh] or cachexia [tiab] or Nutritional Status [Mesh] or Malnutrition [Mesh] or malnutrition* [tiab] or malnourish* [tiab] or nutrition* [tiab] or undernutrition [tiab] or Body Mass Index [Mesh] or body mass index [tiab] or bmi [tiab])) OR (Mobility Limitation [Mesh] or mobilit* [tiab] or Functional* [tiab] or physical* [tiab] or Walking [Mesh] or walk* [tiab] or ambulat* [tiab] or gait [tiab] or Postural Balance [Mesh] or balance [tiab] or hand strength* [tiab] or grip strength* [tiab] or "Muscle Strength"[Mesh] or muscle strength* [tiab] or timed up [tiab] or "Self-Help Devices"[Mesh] or walking aid* [tiab] or wheelchair* [tiab] or "Activities of Daily Living"[Mesh] or ADL [tiab] or IADL [tiab] or living situation [tiab] or community dwelling [tiab] or "Nursing Homes"[Mesh] or nursing home* [tiab] or "Homes for the Aged"[Mesh] or exerci* [tiab] or activit* [tiab] or "Accidental Falls"[Mesh] or fall* [tiab] or "Fractures, Bone"[Mesh] or fracture* [tiab])) OR (sarcopen* [tiab] or "Muscular Atrophy"[Mesh] or atrophy* [tiab] or muscle mass [tiab])) OR ("Quality of Life"[Mesh] or quality of life [tiab] or QOL [tiab] or HRQOL [tiab] or life quality [tiab] or self-rated health [tiab])) OR ("Polypharmacy"[Mesh] or polypharmacy [tiab] or polymedication [tiab] or poly-medication [tiab])) OR ("Urinary Incontinence"[Mesh] or "Fecal Incontinence"[Mesh] or incontinence [tiab]))

Search categories

| *Population* | *Intervention* | *Outcome* |
| --- | --- | --- |
| frail elderly [mesh] or geriatrics [mesh] or very-elder* [tiab] or elder* [tiab] or eldest [tiab] or geriatri* [tiab] or old age* [tiab] or oldest old* [tiab] or very old* [tiab] or senior* [tiab] or septuagenarian* [tiab] or nonagenarian* [tiab] or octogenarian* [tiab] or octagenarian* [tiab] or centenarian* [tiab] or centarian* [tiab] or supercentenarian* [tiab] or older people [tiab] or older subject* [tiab] or older patient* [tiab] or older adult* [tiab] or nursing home [tiab] or 60 year* [tiab] or 65 year* [tiab] or 70 year* [tiab] or 75 year* [tiab] or 80 year* [tiab] or 85 year* [tiab] or 90 year* [tiab] or 95 year* [tiab] or 100 year* [tiab] | 1. Renal replacement therapy: Renal Replacement Therapy [Mesh] or renal replacement [tiab] or kidney replacement [tiab]  2. Dialysis: Renal dialysis [mesh] or dialys* [tiab] or hemodialys* [tiab] or haemodialys* [tiab]  3. Kidney transplantation: Kidney transplantation [mesh] or kidney transplant* [tiab] or renal transplant* [tiab] or kidney graft* [tiab] or renal graft* [tiab] | 1. Cognition: "Cognitive Dysfunction"[Mesh] or cognit* [tiab] or MCI [tiab] or mental* [tiab] or neurocognit* [tiab] or "Mental Status and Dementia Tests"[Mesh] or MOCA [tiab] or MMSE [tiab] or dementia [mesh] or dement* [tiab] or Alzheimer [tiab] or "Delirium"[Mesh] or delirium* [tiab] or "Confusion"[Mesh] or disorientation [tiab] or confusion* [tiab]  2. Mood and anxiety disorders: "Mood Disorders"[Mesh] or mood* [tiab] or affective [tiab] or "Depression"[Mesh] or depressi* [tiab] or neuros* [tiab] or neurot* [tiab] or "Psychotic Disorders"[Mesh] or psychosis [tiab] or psychoses [tiab] or psychotic [tiab] or Anxiety[Mesh] or Anxiety Disorders [Mesh] or anxiety [tiab]  3. Frailty: "Frailty"[Mesh] or frail* [tiab] or vulnerab* [tiab]  4. Nutritional status: "Body Weight"[Mesh] or weight [tiab] or "Weight Loss"[Mesh] or "Cachexia"[Mesh] or cachexia [tiab] or Nutritional Status [Mesh] or Malnutrition [Mesh] or malnutrition* [tiab] or malnourish* [tiab] or nutrition* [tiab] or undernutrition [tiab] or Body Mass Index [Mesh] or body mass index [tiab] or bmi [tiab]  5. Functional status: Mobility Limitation [Mesh] or mobilit* [tiab] or Functional* [tiab] or physical* [tiab] or Walking [Mesh] or walk* [tiab] or ambulat* [tiab] or gait [tiab] or Postural Balance [Mesh] or balance [tiab] or hand strength* [tiab] or grip strength* [tiab] or "Muscle Strength"[Mesh] or muscle strength* [tiab] or timed up [tiab] or "Self-Help Devices"[Mesh] or walking aid* [tiab] or wheelchair* [tiab] or "Activities of Daily Living"[Mesh] or ADL [tiab] or IADL [tiab] or living situation [tiab] or community dwelling [tiab] or "Nursing Homes"[Mesh] or nursing home* [tiab] or "Homes for the Aged"[Mesh] or exerci* [tiab] or activit* [tiab] or "Accidental Falls"[Mesh] or fall* [tiab] or "Fractures, Bone"[Mesh] or fracture* [tiab]  6. Sarcopenia: sarcopen* [tiab] or "Muscular Atrophy"[Mesh] or atrophy* [tiab] or muscle mass [tiab]  7. Quality of life: "Quality of Life"[Mesh] or quality of life [tiab] or QOL [tiab] or HRQOL [tiab] or life quality [tiab] or self-rated health [tiab]  8. Polypharmacy: "Polypharmacy"[Mesh] or polypharmacy [tiab] or polymedication [tiab] or poly-medication [tiab]  9. Incontinence: "Urinary Incontinence"[Mesh] or "Fecal Incontinence"[Mesh] or incontinence [tiab] |

**2. EMBASE**

1. exp dialysis/ or dialys*.ti,ab,kw. or hemodialys*.ti,ab,kw. or haemodialys*.ti,ab,kw.

2. renal replacement therapy/ or renal replacement.ti,ab,kw. or kidney replacement.ti,ab,kw.

3. exp kidney transplantation/ or kidney transplant*.ti,ab,kw. or renal transplant*.ti,ab,kw. or kidney graft*.ti,ab,kw. or renal graft*.ti,ab,kw.

4. 1 or 2 or 3

5. exp cognitive defect/ or cognit*.ti,ab,kw. or MCI.ti,ab,kw. or mental*.ti,ab,kw. or neurocognit*.ti,ab,kw. or exp dementia assessment/ or MOCA.ti,ab,kw. or MMSE.ti,ab,kw. or dement*.ti,ab,kw. or Alzheimer.ti,ab,kw. or delirium/ or postoperative delirium/ or delirium*.ti,ab,kw. or exp confusion/ or disorientation/ or disorientation.ti,ab,kw. or confusion*.ti,ab,kw.

6. exp mood disorder/ or mood*.ti,ab,kw. or affective.ti,ab,kw. or depressi*.ti,ab,kw. or neuros*.ti,ab,kw. or neurot*.ti,ab,kw. or exp psychosis/ or psychosis.ti,ab,kw. or psychoses.ti,ab,kw. or psychotic.ti,ab,kw. or exp anxiety disorder/ or anxiety/ or anxiety.ti,ab,kw. or exp mood disorder assessment/

7. geriatric disorder/ or frailty/ or frail*.ti,ab,kw. or vulnerab*.ti,ab,kw. or vulnerable population/

8. exp body weight/ or weight.ti,ab,kw. or exp malnutrition/ or cachexia.ti,ab,kw. or malnutrition*.ti,ab,kw. or malnourish*.ti,ab,kw. or nutrition*.ti,ab,kw. or undernutrition.ti,ab,kw. or body mass/ or body mass index.ti,ab,kw. or bmi.ti,ab,kw.

9. sarcopen*.ti,ab,kw. or exp muscle atrophy/ or atrophy*.ti,ab,kw. or muscle mass/ or muscle mass.ti,ab,kw.

10. walking difficulty/ or functional status/ or balance disorder/ or balance impairment/ or unsteadiness/ or balance disorder.ti,ab,kw. or balance impairment.ti,ab,kw. or exp walking/ or mobilit*.ti,ab,kw. or Functional*.ti,ab,kw. or physical*.ti,ab,kw. or walk*.ti,ab,kw. or ambulat*.ti,ab,kw. or gait.ti,ab,kw. or balance.ti,ab,kw. or exp hand strength/ or muscle strength/ or hand strength*.ti,ab,kw. or grip strength*.ti,ab,kw. or muscle strength*.ti,ab,kw. or timed up.ti,ab,kw. or exp walking aid/ or wheelchair user/ or walking aid*.ti,ab,kw. or wheelchair*.ti,ab,kw. or daily life activity/ or ADL disability/ or ADL.ti,ab,kw. or IADL.ti,ab,kw. or living situation.ti,ab,kw. or community dwelling.ti,ab,kw. or nursing home/ or nursing home*.ti,ab,kw. or health care facility/ or exp exercise/ or exp physical capacity/ or exerci*.ti,ab,kw. or activit*.ti,ab,kw. or falling/ or fall*.ti,ab,kw. or exp fracture/ or fracture*.ti,ab,kw.

11. "quality of life"/ or exp "quality of life assessment"/ or quality of life.ti,ab,kw. or QOL.ti,ab,kw. or HRQOL.ti,ab,kw. or life quality.ti,ab,kw. or self-rated health.ti,ab,kw.

12. polypharmacy/ or polypharmacy.ti,ab,kw. or polymedication.ti,ab,kw. or poly-medication.ti,ab,kw.

13. exp incontinence/ or incontinence.ti,ab,kw.

14. 5 or 6 or 7 or 8 or 9 or 10 or 11 or 12 or 13

15. frail elderly/ or exp geriatrics/ or institutionalized elderly/ or very-elder*.ti,ab,kw. or elder*.ti,ab,kw. or eldest.ti,ab,kw. or geriatri*.ti,ab,kw. or old age*.ti,ab,kw. or oldest old*.ti,ab,kw. or very old*.ti,ab,kw. or senior*.ti,ab,kw. or septuagenarian*.ti,ab,kw. or nonagenarian*.ti,ab,kw. or octogenarian*.ti,ab,kw. or octagenarian*.ti,ab,kw. or centenarian*.ti,ab,kw. or centarian*.ti,ab,kw. or supercentenarian*.ti,ab,kw. or older people.ti,ab,kw. or older subject*.ti,ab,kw. or older patient*.ti,ab,kw. or older adult*.ti,ab,kw. or nursing home.ti,ab,kw. or 60 year*.ti,ab,kw. or 65 year*.ti,ab,kw. or 70 year*.ti,ab,kw. or 75 year*.ti,ab,kw. or 80 year*.ti,ab,kw. or 85 year*.ti,ab,kw. or 90 year*.ti,ab,kw. or 95 year*.ti,ab,kw. or 100 year*.ti,ab,kw.

16. 4 and 14 and 15

17. limit 16 to conference abstract status

18. 16 not 17

**Appendix 4.** Study characteristics

*Studies in alphabetical order*

| **Paper (first author, year of pulication)** | **Country** | **Study design**  **Centers/source** | **N** | **RRT modality** | **Inclusion criteria** | **Exclusion criteria** | **Age** (years)** | **Outcome(s)** | **Timing of baseline measurement** | **Follow-up period** | **Overall risk of bias** |
| --- | --- | --- | --- | --- | --- | --- | --- | --- | --- | --- | --- |
| Arai et al. 2014^34^ | Japan | Retrospective  Single-center | 202 | HD, PD | Age > 75 |  | 80.4 (4.3) | Functional status | Start of dialysis | Discharge from hospital after start of dialysis; mean (SD) 29 (25.5) days | Serious |
| Basic-Jukic et al. 2008^39^ | Croatia | Prospective  Single-center | 12 | Dialysis | Age > 80 |  | Range 80-92 | Nutritional status | Start of dialysis | 2 years | Serious |
| Bowling et al. 2018^25^ and Plantinga et al. 2017^26^ | USA | Retrospective  Database (USRDS) | 81653 | HD | Age ≥ 67 and < 100 | Missing data | 76.8 (6.5) | Falls | In the year before start of dialysis | 1 year | Moderate |
| De Vecchi et al. 1998^27^ | Italy | Retrospective  Single-center  Subgroup analysis | 63 | PD | Age ≥ 70 |  | 76.5 (4.4) | Functional status, nutritional status | Start of dialysis | 1, 2, 3, 4 and 5 years | Critical |
| Farragher et al. 2020^30^ | Canada | Retrospective  Single-center | 449 | HD | Age > 60; admission to rehabilitation center |  | 73.6 (9.3) | Functional status | Admission to rehabilitation center after start of dialysis | Discharge from rehabilitation center, median (IQR) 43 (33-53) days | Moderate |
| Goto et al. 2019^20^, Goto et al. 2019^21^, and van Loon et al. 2019^22^ | NL | Prospective  Multicenter (n=17) | 160, 187, and 192 respectively | HD, PD | Age ≥ 65 | Terminal non-renal related condition | 75.3 (6.9), 75 (7), and 75 (7) respectively | Falls, functional status, QOL | Start of dialysis | 6 months | Moderate |
| Heldal et al. 2019^23^ and Lønning et al. 2018^24^ | Norway | Prospective  Single-center | 205 and 120 respectively | KT | Age ≥ 65 | Severe cognitive impairment; follow-up < 1 year | 71.5 (4.2), and 71.6 (4.3) respectively | QOL | Before KT (moment of acceptance for KT, six months later or 12 months later) | 2, 6 and 12 months | Moderate |
| Jassal et al. 2009^35^ | Canada | Retrospective  Single-center | 97 | HD, PD | Age ≥ 80 | AKI | 84.5 (3.3) | Functional status | Start of dialysis | Minimum 1 year | Serious |
| Kim et al. 2015^36^ | South Korea | Prospective  Multicenter (n=31)  Subgroup analysis | 410 | HD, PD | Age ≥ 65 | AKI; missing data | HD: 72.2 (5.4). PD: 70.3 (4.4) | QOL, mood and anxiety disorders | Start of dialysis | 1 year | Serious |
| Kurella Tamura et al. 2009^37^ | USA | Retrospective  Database (USRDS) | 3702 | HD, PD | Nursing home resident | Missing data | 73.4 (10.9) | Functional status | 0 to 3 months before start of dialysis | 3 months and 1 year | Moderate |
| Lægreid et al. 2014^38^ | Norway | Retrospective  Database (NRR) | 233 | HD, PD | Age ≥ 75 | History of HD or KT; deceased or not on dialysis at study end date | 78.4 (4.2) | Nutritional status | Start of dialysis | Mean (SD) 36.7 (28.7) months | Critical |
| Lai et al. 2018^33^ | Italy | Prospective  Single-center  Subgroup analysis | 29 | PD | Age ≥ 65; clinically stable | Cancer, liver disease, HIV; missing data | 74.1 (6.4) | QOL | Start of dialysis | 2 years | Serious |
| Lee et al. 2017^31^ | South Korea | Prospective  Single-center | 46 | HD | Age ≥ 65 | Cancer (diagnosis <3 months before start of dialysis), AKI | Median (IQR): 71.5 (67.8-76.3) | Frailty, functional status, nutritional status | Start of dialysis | 1 year | Serious |
| López-Montes et al. 2020^32^ | Spain | Prospective  Single-center | 117 | HD | Age ≥ 70 | Active infectious disease or cancer; history of RRT | 78.1 (4.1) | Cognition, functional status, mood and anxiety disorders | Start of dialysis | 1 year | Serious |
| McAdams-DeMarco et al. 2015^28^ | USA | Prospective  Single-center  Subgroup analysis | UNK | KT | Age ≥ 65 | < 1 hospital visit after KT | UNK | Frailty | KT | 3 months | Moderate |
| Ortega et al. 2009^29^ | USA | Prospective  Multicenter (n=4)  Subgroup analysis | UNK | KT | Age > 60 |  | UNK | QOL | Before KT | 1 year | Serious |

Abbreviations: ADL = activities of daily living; AF = atrial fibrillation; AKI = acute kidney injury; BMI = body mass index; HD = hemodialysis; HIV = human immunodeficiency virus; IADL = instrumental activities of daily living; IQR = interquartile range; KT = kidney transplantation; MI = myocardial infarction; NA = not applicable; NIHRD = Taiwan's National Health Insurance Research Database; NL = the Netherlands; NRR = Norwegian Renal Registry; P = peritoneal dialysis; ROBINS-I = Risk Of Bias In Non-randomized Studies of Interventions; QOL = quality of life; RRT = renal replacement therapy; UNK = unknown; USA = United Stated of America; USRDS = United States Renal Data System.

* only part 2: cohort study

** Presented as mean (standard deviation) unless stated otherwise.
